# Screen-Printed Silver/Silver Chloride Electrodes Inhibit Alcohol Oxidase Activity

**DOI:** 10.1149/2754-2726/ace5a9

**Published:** 2023-07-18

**Authors:** Bob M. Lansdorp, Peter Lamberg, Rashad Hamid

**Affiliations:** 1 Milo Sensors, Inc. Santa Barbara, CA, 93101, United States of America

## Abstract

Silver/silver chloride (Ag/AgCl) is ubiquitous in the field of electrochemical biosensing due to its suitability as a reference electrode material. However, we recently discovered that screen-printed Ag/AgCl ink has a detrimental effect on Alcohol Oxidase enzyme stability. We performed an optical absorbance assay to isolate the interaction of enzyme and electrode to discover a surprisingly strong inhibition effect. The halftime of enzymatic activity was reduced from nearly 1 week in buffer to 10 h in the presence of the Ag/AgCl electrode. We expect this discovery to have broad implications on enzymatic biosensors that use Ag/AgCl as reference electrode material.

In biosensors, it is imperative that the electrical potential of a sensor surface of interest can be accurately determined or controlled. To this end it is customary use a reference electrode whose potential is relatively stable, and then to measure the relative potential with respect to that reference electrode.^1^ The silver/silver-chloride (Ag/AgCl) reference electrode has been widely used in biosensors due to its simple design, ease of manufacture, and environmental friendliness.^2^ A search on PubMed for “Ag/AgCl” returned 4990 results.

The Ag/AgCl electrode relies on the equilibrium between dissolved chloride ions Cl^−^, chloride that binds with silver (Ag) metal to form silver chloride (AgCl) on a Ag electrode. The rapid exchange of chloride between chloride in liquid and solid makes Ag/AgCl a favorable choice for a reference electrode material. Ag/AgCl electrodes typically hold a reference potential of between +0.29 V and +0.20 V vs Standard Hydrogen Electrode in typical Cl^−^ concentrations between 0.1 M and saturation, respectively.^3^ Often, a three-electrode measurement setup is used, which consists of a working electrode with a surface of interest, a counter electrode to provide a counter-current, and reference electrode to measure or control the potential. In case the measured electrical current is relatively low such that the ohmic drop over the reservoir is low as compared to what would be considered a significant voltage excursion, the counter and reference electrode can be combined into a single quasi-reference electrode. Although attention has been given to the stability of a quasi-reference electrode potential as a function of analyte concentration according to traditional electrochemistry methods such as the Nerst equation,^4^ to our knowledge there has been only one mention of potential detrimental impact that Ag/AgCl electrodes can have on enzyme stability.^5^

There are many methods available to manufacture Ag/AgCl electrodes. A silver wire can be immersed in bleach,^6^ or silver can be chlorinated utilizing an ion exchange reaction such as immersion in FeCl_3_, or electrochemical coating through anodization.^4^ However, a typical manufacturing process is to screen-print using an ink consisting of silver nanoparticles (AgNPs) mixed with silver chloride (AgCl) powder, with a solvent, mechanically pushing said ink through a fine metal mesh and onto a substrate, removing the metal mesh, and baking the ink onto the substrate to yield Ag/AgCl electrodes. The screen-printing process is highly economical, and as a result, screen-printed Ag/AgCl electrodes are ubiquitous in the field of biosensors.

Enzymes provide a highly selective means of detecting analytes even in the presence of background interferents, and enzymatic biosensors are seeing increased application in the field. However, enzyme stability is a common problem that limits the continuous operation of biosensors. AgNPs are commonly used in anti-microbial applications,^7^ but relatively few studies have specifically examined the influence of AgNPs on enzyme stability. One study found that Jack Bean Urease (JBU) activity could be inhibited by AgNPs.^8^ Another study found that silver ions (Ag+) could leach out of an Ag/AgCl electrode to inhibit creatine amidinohydrolase.^5^ Other studies have found that Glucose Oxidase could be inhibited by freshly prepared AgNPs, although there was a complicated enhancement effect as the AgNPs were aged.^9–12^

In a previous work, we showed how Alcohol Oxidase (AOD) could be combined with a Prussian Blue working electrode and a Ag/AgCl quasi-reference electrode to create a novel wearable alcohol biosensor.^13^ We observed that active life of the sensor seemed to be limited to less than 24 h, and that the lifetime was limited by enzyme inhibition, but the mechanism behind the limited enzyme life was not clear. Here, we provide evidence for the root cause of the degradation: AOD is destabilized by the presence of a Ag/AgCl reference electrode.

## Experimental

We performed an electrochemical experiment as previously described^13^ where the presence of active enzyme was continuously monitored amperometrically in the presence of ethanol flow. A screen-printed electrode was manufactured by Boyd, utilizing a Ag/AgCl ink (Dupont^®^ 5874), screen printed onto a polyester substrate utilizing a 325SS mesh. From a Technical Data Sheet,^14^ we can see that the ink consists of silver chloride at 35%, silver nanoparticles at 65%. The silver chloride had a surface area of approximately 5.6 mm^2^. The electrode was placed in an injection-molded polypropylene clamshell and electrical current was measured at an applied potential of +93 mV vs the pseudo reference electrode in a custom made potentiostat. The sample was allowed to baseline for at least 2 h, after which buffer solution containing 25 mM ethanol was flown over a semi-permeable membrane on top of the sensor continuously. The enzyme is a stabilized lyophilized AOD powder containing a proprietary blend of polysaccharides (Sun Chemical) that remains stable for up to two years while in a dry phase. The enzyme was hydrated utilizing an agarose hydrogel at 0.25 wt% in pH 7.4 Phosphate Buffered Saline at 100 mM.

We used an optical absorbance 2,2′-azino-bis(3-ethylbenzothiazoline-6-sulfonic acid) (ABTS) Assay^15^ to isolate the influence of a screen-printed Ag/AgCl electrode on AOD enzyme activity. The working principle of the ABTS Assay is that Ethanol is processed by a rate-limiting concentration of AOD to yield Hydrogen Peroxide, which was converted by an excess of Horseradish Peroxidase (HRP) and readily available ABTS substrate to yield oxidized ABTS, which is measurable as a change in optical absorbance that is proportional to the AOD activity. AOD Solution consisting of 0.3 mg of AOD dissolved in 100 *μ*L of 100 mM Phosphate Buffered Saline, was left in polypropylene Eppendorf tube for varying lengths of time, both with and without a screen-printed Ag/AgCl electrode immersed in the AOD solution. An ABTS Assay Solution was made, consisting of 100 mM Phosphate Buffer at pH 7, 1 mg ml^−1^ of ABTS powder (taken from freezer) and 2.63 U of HRP was placed in an optical cuvette and monitored at a wavelength of 405 nm with approximately one measurement per three seconds using a Spectronic 1001 +UV/Vis spectrophotometer (Milton Roy) that was connected via a serial-USB connector to a PC. Data was logged using a custom Labview data acquisition program. After a 30 min baseline period, the 10 *μ*L of AOD solution was added. This was repeated for varying AOD solution incubation times including t = 0, 5, 20, 25 and 45 h.

## Results and Discussion

**Figure 1.**
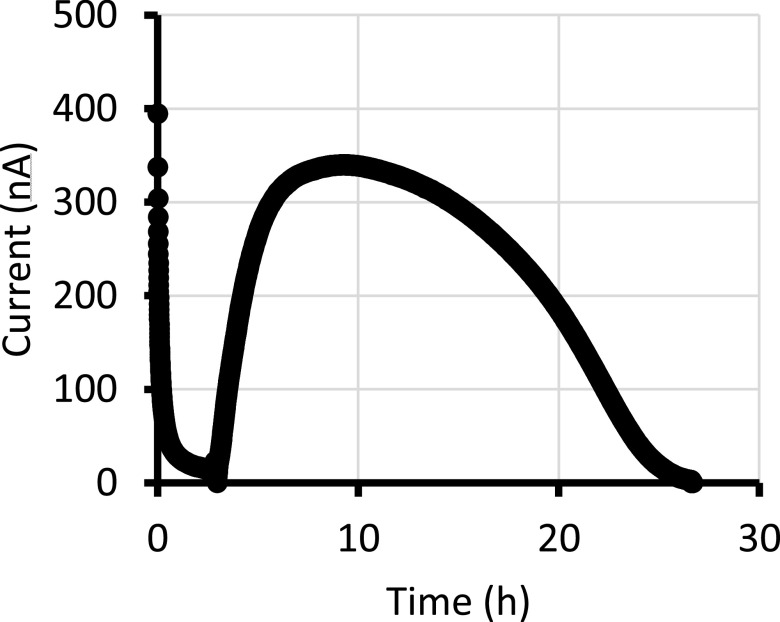
A chrono-amperometric curve of AOD in an electrochemical cell kept at +93 mV vs AgCl. The cell contained 35 *μ*L Phosphate buffered saline set into a 0.25 wt% Agarose gel. The working electrode is screen-printed Prussian blue and the pseudo-reference electrode screen-printed AgCl.

In the electrochemical assay, the measured electrical current decayed initially to a baseline current. We have shown previously that this initial current decay to baseline is related to Prussian Blue and its interaction with Alcohol Oxidase (Fig. 3 and Supplemental Figs. S3 and S4 from^13^). Upon ethanol addition, we observed that the electrical current increased rapidly from baseline to plateau (Fig. 1). The current stayed near a plateau value for approximately 10 h to the membrane-diffusion mass-transfer limited current, but then decayed back to baseline due to enzyme inhibition. We note that although the amperometric current amplitude is qualitatively related to residual enzyme activity, it is not linearly proportional to residual enzymatic activity due to the mass-transfer limitation of the diffusion-limiting membrane. We previously demonstrated sensor degradation was not caused by Prussian Blue deactivation, nor Ag/AgCl electrode drift, nor by membrane fouling, but by enzyme inhibition (see Supplemental S5 from^13^).

In an effort to determine the root cause of enzyme inhibition, in this work we have utilized an ABTS assay to isolate and simplify the system (no electrical measurements, no electrodes, no diffusion-limiting membrane, etc.). The slope of the absorbance vs time in the first 10 min after ethanol addition, after subtracting off the pre-ethanol-addition baseline drift slope, was used to calculate residual enzyme activity.^16^ The slope of absorbance vs time could be converted into enzyme activity (*μ*M/s) by using Beer–Lambert law with an extinction coefficient of 36.8 mM^−1^ cm^−1^ at 405 nm for ABTS. Each experiment was performed in triplicate, with data points representing the average time and activity, with an activity error bar calculated as the standard deviation of the three measurements.

**Figure 2.**
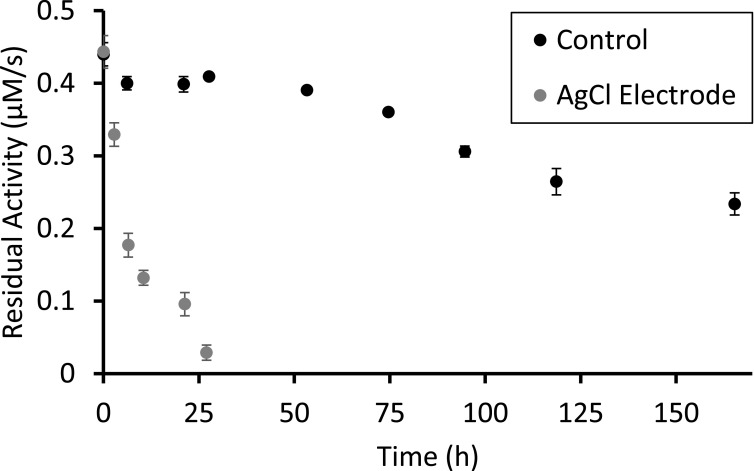
Alcohol Oxidase activity, measured using an optical absorbance assay, as a function of time incubated with destabilizing agents. Each data point is measured at 405 nm after baselining for 30 min and measuring activity after ethanol addition for 10–20 min.

The results are plotted in Fig. 2. Enzymes dispersed in the PBS buffer left in cuvettes were found to still be active, even after a week of storage (see Fig. 2 - Control). When a screen-printed Silver/Silver-Chloride electrode was added to the cuvette with 100 *μ*L of buffer, and left for 24 h, the enzyme activity had significantly degraded within 12 h (Fig. 2 - AgCl Electrode). This surprising phenomenon of AgCl degrading AOD is therefore the root cause of the limited lifetime of the electroenzymatic ethanol sensor, since the AOD solution in the electrochemical sensor is in constant contact with the Ag/AgCl electrode, and we have conclusively demonstrated a detrimental interaction. We anticipate that this result could have broad implications for the biosensor community, and specifically any group that aims to use an enzymatic sensing modality together with a Ag/AgCl electrode.

We have conclusively demonstrated that the presence of a screen-printed Ag/AgCl electrode inhibits the activity of AOD enzyme. We suspect that silver nanoparticles are leaching form the screen-printed electrode, and that these particles are destabilizing the AOD through binding interactions with the protein surface, since cysteine residues have been shown to bind to silver.^5^ However, additional work is needed to reveal the precise mechanism by which Ag/AgCl electrode destabilizes AOD, whether it is due to destabilization of enzyme structure or subunits, by silver particles that have leached into the solution, adsorption of the enzyme onto the electrode surface, or other mechanisms. Future studies that measure the rate that AgNPs and Ag+ ions are leached into solution by screen-printed electrode surfaces would be of interest. In our setup, we utilized a two-electrode setup with a relatively large surface area Ag/AgCl quasi-reference electrode (5.6 mm^2^ surface area). It is possible that a three-electrode setup utilizing a smaller surface area Ag/AgCl electrode or one with a porous membrane would be less vulnerable to enzyme inhibition by the electrode. Alternative electrode materials such as copper-copper sulphate or saturated calomel in a three-electrode setup are expected to provide similar results to Ag/AgCl with glass frit in a three-electrode configuration since we expect that any of these tests would overcome all three primary hypothesized mechanisms of destabilization. Scanning electron microscope (SEM) images of the Ag/AgCl electrode surface before and after 48 h of enzymatic activity could help provide insights into the role of Ag/AgCl particle leaching. Future work to overcome the inhibition of AOD by Ag/AgCl would be of interest to those developing enzymatic alcohol biosensors.

Our discovery that Ag/AgCl inhibits AOD has broad implications to the field of biosensors. When enzymes are coupled with an electrochemical measurement, it is imperative that the functionality of the enzyme is confirmed, even after variable incubation times with the other electrochemical components. To our knowledge, our study is only the second to demonstrate a link between Ag/AgCl ink and enzyme inhibition, but we expect to see this trend repeated as more biosensors utilizing enzymes together with Ag/AgCl are developed.

## Conclusions

Our ABTS Assay result shows that the presence of a Ag/AgCl electrode significantly destabilizes AOD from one week to a half-life of less than 10 h. The destabilizing effect of Ag/AgCl may carry over into many other enzymes, given that a similar effect has been seen with JBU, which has a different structure and mechanism compared to AOD.^8,17^ Developers of enzymatic electrochemical biosensors are cautioned to verify enzyme activity in the presence of a screen-printed Ag/AgCl electrode, especially if the biosensor is intended to be used over the course of a day or more, or the electrode is stored in the same solution as the enzyme.
